# Acceptability, Engagement, and Effects of a Mobile Digital Intervention to Support Mental Health for Young Adults Transitioning to College: Pilot Randomized Controlled Trial

**DOI:** 10.2196/32271

**Published:** 2021-10-14

**Authors:** Brian Suffoletto, Tina Goldstein, Dawn Gotkiewicz, Emily Gotkiewicz, Brandie George, David Brent

**Affiliations:** 1 Department of Emergency Medicine Stanford University Palo Alto, CA United States; 2 Department of Psychiatry University of Pittsburgh Pittsburgh, PA United States; 3 University of Delaware Newark, DE United States

**Keywords:** college, mental health, self-management, digital intervention, mHealth

## Abstract

**Background:**

The transition from high school to college can exacerbate mental health problems in young adults yet barriers prevent seamless mental health care. Existing digital support tools show promise but are not yet designed to optimize engagement or implementation.

**Objective:**

The goal of the research was to test acceptability and effects of an automated digital Mobile Support Tool for Mental Health (MoST-MH) for young adults transitioning to college.

**Methods:**

Youths aged 18 years and older with a current mental health diagnosis preparing to transition to college (n=52; 85% female [45/52], 91% White [48/52]) were recruited from a primary care (n=31) and a mental health clinic (n=21). Participants were randomized 2:1 to either receive MoST-MH (n=34) or enhanced Usual Care (eUC; n=18). MoST-MH included periodic text message and web-based check-ins of emotional health, stressors, negative impacts, and self-efficacy that informed tailored self-care support messages. Both eUC and MoST-MH participants received links to a library of psychoeducational videos and were asked to complete web-based versions of the Mental Health Self-Efficacy Scale (MHSES), College Counseling Center Assessment of Psychological Symptoms (CCAPS), and Client Service Receipt Inventory for Mental Health (C-SRI) monthly for 3 months and the Post-Study System Usability Scale (PSSUQ) at 3-months.

**Results:**

MoST-MH participants were sent a median of 5 (range 3 to 10) text message check-in prompts over the 3-month study period and 100% were completed; participants were sent a median of 2 (range 1 to 8) web-based check-in prompts among which 78% (43/55) were completed. PSSUQ scores indicate high usability (mean score 2.0). Results from the completer analysis demonstrated reductions in mental health symptoms over time and significant between-group effects of MoST-MH compared to eUC on depressive symptom severity (d=0.36, 95% CI 0.08 to 0.64). No significant differences in mental health self-efficacy or mental health health care use were observed.

**Conclusions:**

In this pilot trial, we found preliminary evidence that MoST-MH was engaged with at high rates and found to be highly usable and reduced depression symptoms relative to eUC among youth with mental health disorders transitioning to college. Findings were measured during the COVID-19 pandemic, and the study was not powered to detect differences in outcomes between groups; therefore, further testing is needed.

**Trial Registration:**

ClinicalTrials.gov NCT04560075; https://clinicaltrials.gov/ct2/show/NCT04560075

## Introduction

Mental health disorders are common among young adults. Internationally, 20% of college students meet criteria for a mental health diagnosis, and 83% of these individuals have onset prior to college matriculation [1]. Nationally, 31% of US college students report having a mental health diagnosis [2], and rates of mental health disorders in young adults have been increasing [3]. Mental health disorders can have significant negative impact on young adults including lower grade point averages and higher rates of college attrition [4]. Mental health disorders can also put a young adult at greater risk for physical health problems [5] and suicide [6].

The transition from high school to college is a critical period where mental health support is lacking yet urgently needed. New stresses related to academics, finances, and relationships are heightened upon college initiation [7]. These stressors can worsen mental health symptoms and precipitate suicidal ideation [8]. Concurrently, the transition from pediatric to adult health services that frequently occurs at this juncture means that young adults often leave the providers with whom they have built longitudinal trusting relationships. Compounding this are stigma [9], scheduling difficulties [10], and lack of in-person resources to accommodate the mental health needs of all students [11,12]. For these reasons, novel approaches to supporting college students with mental health self-management are urgently needed.

Digital technology could assist young adults who are transitioning to college by providing rapid and efficient access to mental health support. In a systematic review of 19 studies (n=11 randomized controlled trials [RCTs]) of digital mental health interventions for college students, findings suggest that they can be effective for improving depression, anxiety, and psychological well-being [13]. Another recent systematic review focused on mobile mental health interventions for college students found that they reduce psychological symptomatology associated with stress, depression, anxiety, and general student mental health [14]. Despite these promising findings, most of these interventions addressed only a single mental health disorder. Also, many of these interventions incorporated some form of human support, making broad and cost-efficient implementation problematic. Finally, it is widely understood that, to date, longitudinal engagement with mental health digital interventions has been poor [15], limiting durability of effects.

**Figure 1:**
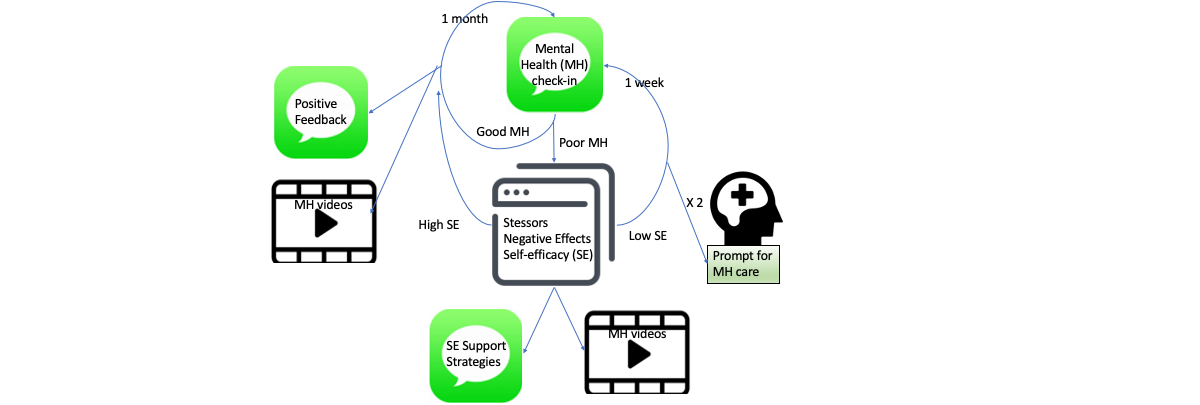
Mobile Support Tool for Mental Health design diagram.

To address these barriers, we developed an automated Mobile Support Tool for Mental Health (MoST-MH). MoST-MH was iteratively designed and refined by a multidisciplinary team with expertise in psychology, psychiatry, primary care, and digital interventions with integral feedback from a college student ambassador. MoST-MH was intended to provide support independent of mental health diagnosis type (ie, transdiagnostic) and designed to minimize the burden of intensive digital interactions using a stepwise algorithm which adapts frequency of interaction to the needs of the youth. MoST-MH is an ecological momentary intervention [16] in that it provides support in the context of a young adult’s current state and needs. Specifically, MoST-MH incorporated periodic text message mental health check-ins, triggering web-based check-ins (when mental health was rated low) to understand stressors, negative effects, and self-efficacy, which informed self-efficacy support strategies and prompted links to psychoeducational videos focused on college and mental health. Figure 1 outlines the design of MoST-MH.

In this paper, we present pilot randomized trial findings of MoST-MH where we examined intervention engagement, acceptability, and estimates of effects of MoST-MH compared to enhanced usual care (eUC). We hypothesized that youth would engage with MoST-MH at high rates over the first 3 months of college and that they would report high levels of usability. We also hypothesized that youth who received MoST-MH, as compared with youth who receive eUC, would report greater mental health self-efficacy, lower symptom severity, and higher rates of follow-through with mental health care at 3 months.

## Methods

### Study Design

We conducted a pilot randomized trial among youth with a current mental health disorder or recent mental health care preparing to transition to college. Design and a priori hypotheses were registered at ClinicalTrials.gov (NCT04560075). As this was a pilot study, we were not powered to detect significant differences in mental health outcomes between groups. All participants completed written informed consent. Study investigators and outcome assessors were blinded to allocation to treatment arms. All procedures were approved by the institutional review board at the University of Pittsburgh.

### Participants

Participants were recruited from one primary care (n=31) and one mental health clinic (n=21) in Pittsburgh, PA, from August to October 2020. We chose to recruit from health care sites because we view the ultimate implementation to be initiated by care providers who are able to identify individuals with mental health needs prior to leaving for college. The youth’s care providers identified potentially eligible youth and asked the youth about interest in participating in the study; interested youth were texted or emailed a web link that provided information about the study. If they were interested, they contacted the research team via telephone, where enrollment criteria were confirmed. Inclusion criteria included age 18 years or older, current mental health diagnosis documented in their electronic medical record or received mental health services within 3 months per self-, parent-, or clinician-report, graduated high school, plan to attend college or higher education within 6 weeks, and own a personal mobile phone with text messaging. We excluded non-English–speaking individuals given that intervention materials were in English only.

### Randomization

We used block randomization whereby two-thirds of participants were randomly assigned to receive MoST-MH and one-third to receive eUC. Blocks balanced the groups based on recruitment site. Random assignment allocation occurred following completion of baseline assessments.

### MoST-MH Intervention

MoST-MH aimed to enhance an individual’s mental health by raising awareness of current symptoms, stressors, and impact of stressors and boosting self-efficacy by prompting evidence-based strategies taken from positive psychology, cognitive behavioral therapy, and dialectical behavioral therapy. MoST-MH used text messaging as the primary communication modality given its ubiquity and preferential role for communication among youth [17] as well as its proven effectiveness to deliver other forms of health support [18]. MoST-MH incorporated web-based check-ins when mental health was rated as suboptimal to collect more detailed information to help guide tailoring of support and because lengthy checklists would have been cumbersome using text messaging. The MoST-MH intervention software was run by the Office of Academic Computing at the University of Pittsburgh Medical Center.

Upon allocation, MoST-MH participants were prompted to text a unique keyword to a study phone number to initiate the program. Once program was initiated, participants received a series of welcome messages describing what to expect over the intervention period and ways to reduce breach of privacy (eg, “Welcome to MoST-MH. Over the next 3 months we’ll be checking in by text message. Set up a password on your phone and erase messages you do not want anyone to see after reading them”). Participants were instructed that they could drop out of the MoST-MH program at any time by texting Quit.

Starting on the day of enrollment, MoST-MH participants received mental health check-ins via text message: “How would you rate your emotional health this past week?” If they replied excellent, very good or good, they received a positive feedback text message and link to video library. The brief 2-minute videos were created by the study team and included psychoeducation about mental health self-care during college. If they replied fair or poor, they were sent a link to complete a web-based check-in. When the web link opened, a page displayed a checklist of common stressors [7] and negative effects. The student was asked a self-efficacy question: “To what extent do you feel you can manage your stressors and negative effects with supports and skills you have?” If they reported high self-efficacy (completely), they received positive feedback and a web link to a library of mental health videos and the program was timed to check in with them in a month.

**Figure 2:**
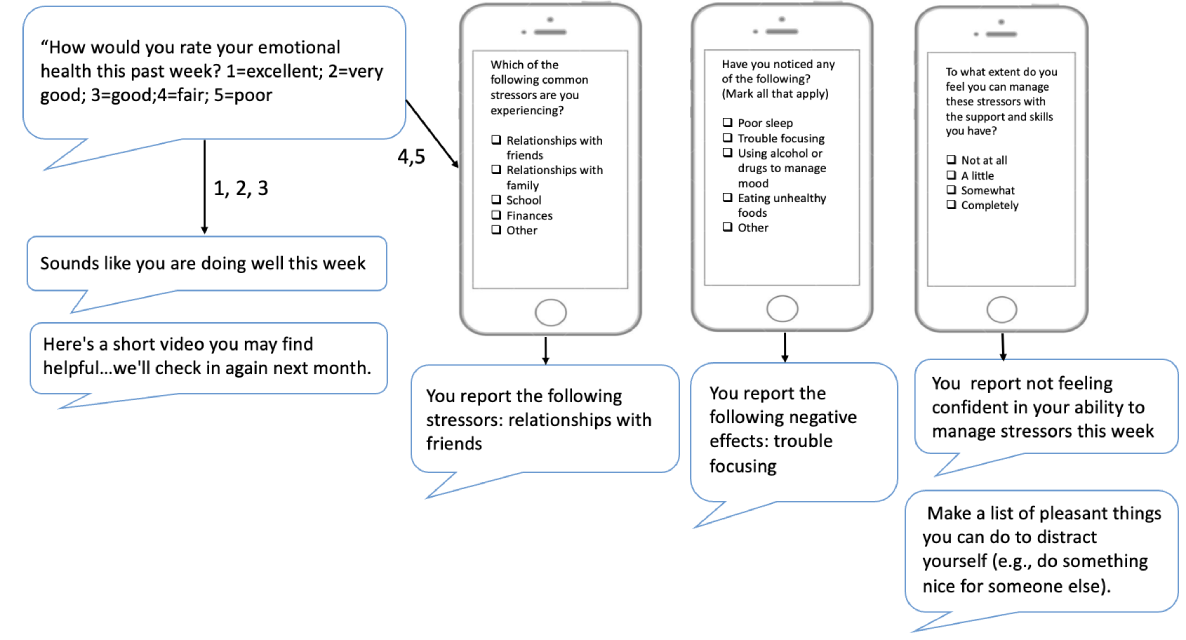
Mobile Support Tool for Mental Health example exchange.

If they reported low self-efficacy (somewhat, a little, or not at all), they received a text message from a skills library and the link to the videos and were asked if it was ok to check in next week. On subsequent MoST-MH check-ins, their reports of stressors and negative effects were compared to the prior assessment and feedback incorporated relative improvement or unresolved stressors/effects. If the ability to self-manage stressors or negative effects was still reported as suboptimal, the individual was prompted to consider making an appointment for seeking mental health care: “Your doctor or another health professional can help. Would you be willing to reach out to them to set up an appointment?” If they were willing, they were provided with a link to resources to assist. Throughout all program queries, missing responses were reprompted once only. To ensure safety, if an individual reported poor mental health and low self-efficacy 2 weeks in a row, they were prompted to seek formal mental health care. Figure 2 demonstrates a sample communication exchange.

### Enhanced Usual Care

The eUC participants received a web link to a library of the same psychoeducational videos provided to the MoST-MH group. eUC received no text message or web-based mental health check-ins.

### Measures

MoST-MH engagement was assessed using text messaging and web-based responses. Mental health symptom severity and health care use were assessed using self-report measures collected monthly for 3 months. Each monthly assessment battery was estimated to take 15 minutes to complete and were completed on a smartphone, laptop, tablet, or desktop. Participants in both groups were sent text message reminders every 3 days up to 3 times prompting them to complete their web-based follow-up assessment batteries. Participants were eligible to receive a total of $100 for participation in the study, including $20 for completing each monthly assessment battery. Participants were not compensated for completing text messages nor web-based assessments.

MoST-MH usability was measured via the Post-Study System Usability Scale (PSSUQ) [19] at 3 months only. The PSSUQ includes 19 items, each rated on a 7-point Likert-type scale ranging from 1 (strongly agree) to 7 (strongly disagree). The psychometric factors of the PSSUQ are overall usability, system usefulness, information quality, and interface quality. The lower the score (to a limit of one), the higher the perceived usability.

Mental health self-efficacy was measured using the 6-item self-report Mental Health Self-Efficacy Scale (MHSES) [20], which asks participants to rate each statement on a 10-point Likert scale ranging from 1 (not at all confident) to 10 (totally confident) whereby higher scores indicate higher self-efficacy: “On an average day in the next month, how confident are you that… (1) You can keep your stress, anxiety, or depression from interfering with the things that you want to do? (2) You can do the different tasks and activities needed to manage your stress, anxiety, or depression so as to reduce your need to see a doctor? (3) You can do things other than just taking medicine to reduce how much your stress, anxiety, or depression affects your everyday life? (4) You can make your days at least moderately enjoyable? (5) You will have moderate amounts of time where you do not experience stress, anxiety, or depression? (6) You will be able to effectively manage any stress, anxiety, or depression that you do experience?”

Symptom severity was measured using the College Counseling Center Assessment of Psychological Symptoms (CCAPS) [21] which has 62 items with 8 distinct subscales of psychological symptoms for college students: (a) depression (13 items), (b) generalized anxiety (9 items), (c) social anxiety (7 items), (d) academic distress (5 items), (e) eating concerns (9 items), (f) family distress (6 items), (g) hostility (7 items), and (h) substance use (6 items). Items are scored on a 5-point Likert scale from 0 (not at all like me) to 4 (extremely like me), whereby higher scores indicate higher symptom severity.

Mental health treatment use was measured using the brief self-report Client Service Receipt Inventory for Mental Health (C-SRI) [22] including outpatient, inpatient, and medication management services.

### Data Analysis

To determine whether any significant differences between groups existed at baseline, independent *t* tests were conducted on continuous baseline variables (ie, age, MHSES, CCAPS), and chi-square analyses were conducted on categorical or nominal variables (ie, gender, race, ethnicity, college plans and living situation, site of recruitment, C-SRI mental health care). We tested the hypothesis that youth would engage with MoST-MH at high rates (>80% response rate) by calculating text message and web check-in completions within and between individuals. We tested the hypothesis that youth would report high levels of usability with MoST-MH (mean PSSUQ rating ≤2) by computing PSSUQ ratings at 3-month follow-up. We explored the effect of MoST-MH as compared with eUC on mental health self-efficacy (MHSES), symptom severity (CCAPS), and mental health care services use (C-SRI) using mixed effect (general estimating equation [GEE]) models. Mixed effects models using GEE are recommended for analysis of repeated-measures data and can properly account for missing data [23]. To understand for whom the intervention may work better or worse, we explored associations between patient factors (sex, race, planned college attendance, baseline CCAPS scores) and engagement, usability, and mental health outcomes using univariate GEE models. Primary analyses were conducted using listwise deletion. For sensitivity analyses, we conducted intention-to-treat analyses using multiple imputation procedures where missing CCAPS outcome data were assumed to be missing at random. A simulated dataset with 20 imputed outcomes was generated. All analyses were conducted using Stata (version 15.0, StataCorp LLC).

## Results

### Overview

**Figure 3:**
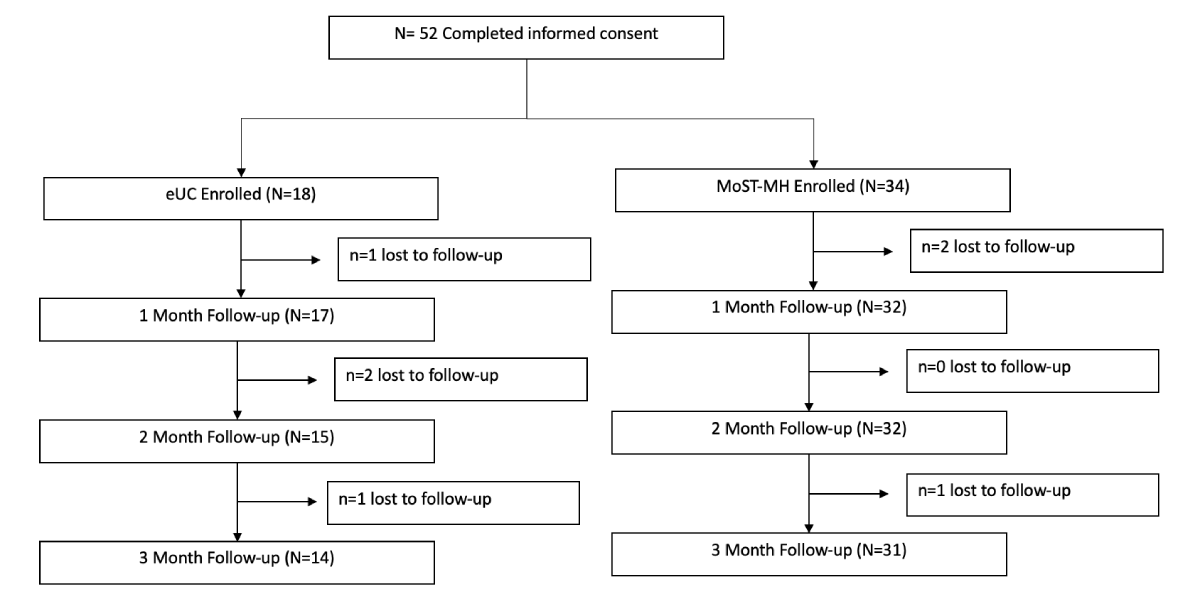
Consolidated Standards of Reporting Trials diagram. eUC: enhanced usual care; MoST-MH: Mobile Support Tool for Mental Health.

Figure 3 shows the participant flow throughout the study. A total of 98 youths were referred to the study, 73 were reached for screening, and 52 completed informed consent. The resultant sample was randomized via computer algorithm to receive either instructions to initiate MoST-MH (n=34) or eUC (n=18) after completion of online questionnaires at baseline. A total of 49/52 (94%) of participants completed 1-month follow-up assessment batteries, 47/52 (90%) completed 2-month follow-up assessment batteries, and 45/52 (87%) completed 3-month follow-up assessment batteries. There were no differences in attrition by sex, race, college living plans, or treatment arm.

### Participants

Table 1 shows the demographics of the sample of MoST-MH and eUC participants. Most identified as female (45/52, 85%) and White (48/52, 91%). The majority of the sample (45/52, 84%) was planning on attending a 4-year college, although with a wide variety of plans for college living situation. There was a higher percentage of White youths and individuals planning on living on campus in the MoST-MH arm compared to eUC. For that reason, we included race and planned college living situation (in addition to sex) as covariates in all models.

**Table 1:** Participant characteristics.

Characteristic			eUC^a^ (n=18)		MoST-MH^b^ (n=34)		*P* value	
Age (years), mean (SD)			18.7 (0.48)		18.7 (0.42)		.47	
Sex, female, n (%)			18 (100)		27 (79)		.08	
**Race, n (%)**								.04
	White	15 (83)		33 (97)		—^c^		
	Black	0		1 (3)		—		
	More than one	3 (17)		0		—		
Hispanic, n (%)			1 (6)		2 (6)		.53	
**College plans, n (%)**								.48
	4 year	15 (83)		30 (88)		—		
	Community	2 (11)		1 (3)		—		
	Professional/trade	1 (6)		1 (3)		—		
	Other	0		2 (6)		—		
**Plans for living, n (%)**								.02
	On campus, dorms (roommate)	6 (33)		25 (74)		—		
	On campus, dorms (by self)	3 (17)		2 (6)		—		
	Off campus	3 (17)		1 (3)		—		
	At parents	6 (33)		6 (18)		—		
**Site of recruitment, n (%)**								.66
	Primary care	10 (56)		21 (62)		—		
	Mental Health clinic	8 (44)		13 (38)		—		

^a^eUC: enhanced usual care.

^b^MoST-MH: Mobile Support Tool for Mental Health.

^c^Not applicable.

### MoST-MH Engagement

Participants were sent a median of 5 (range 3-10) text message check-ins over the study period (depending on their risk level), and 100% of the text message queries were completed. The 21 participants who reported poor MH via text message at least once over the study period received a median of 2 (range 1 to 8) web-based check-ins. Of the 55 times when a web check-in was prompted, 43 (78%) were completed. We did not find that sex, race, or college living plans were significantly associated with web check-ins. No MoST-MH participants dropped out (ie, texted Quit).

The median number of stressors reported per check-in was 2 (range 0 to 5); the median number of negative effects reported per check-in was 3 (range 0 to 8). There were higher mean stressors reported in female compared to male participants (beta=0.49, 95% CI 0.21to 0.76). The most common stressors were related to school and finances; the most common negative effects were feeling worn out and low motivation. Table 2 shows the percentage of check-ins with a given stressor and negative effects reported. Self-management self-efficacy was rated as high 9% (4/43) of the time and low 91% (39/43) of the time. We did not find that patient factors (ie, sex, race, college plans) were significantly associated with self-efficacy. Higher baseline anxiety scores were associated with lower self-efficacy (beta=–0.32, 95% CI –0.55 to –0.08).

**Table 2:** Frequency of reported stressors and negative effects for Mobile Support Tool for Mental Health participants (n=34).

Characteristic			Value, n (%)
**Stressors**			
	School	22 (51)	
	Finances	16 (37)	
	Relationships with friends/roommates	12 (28)	
	Relationships with family	11 (26)	
	Romantic relationships	9 (21)	
	Other	3 (7)	
**Negative effects**			
	Feeling worn out	28 (65)	
	Low motivation	24 (56)	
	Trouble focusing on schoolwork	22 (51)	
	Poor sleep	20 (47)	
	Unhealthy eating habits	18 (42)	
	Feeling like you are overreacting	14 (33)	
	Feeling like you don’t have people to talk to	13 (30)	
	Using alcohol or drugs to manage emotions	5 (12)	
	Other	1 (2)	

### Intervention Usability

Using the PSSUQ completed at the 3-month follow-up, overall mean usability score was 2.0 (SD 1.6). For subscale rating, mean score for system usefulness was 1.9 (SD 1.7), information quality was 2.2 (SD 1.5), and interface quality was 1.9 (SD 1.7). We did not find that patient factors (ie, sex, race, college plans) or baseline mental health scores were significantly associated with usability.

### Mental Health Self-Efficacy

Using the MHSES, there were no significant improvements in either treatment arm over time for mental health self-efficacy as measured. In GEE analysis, there were no significant time by treatment effects of MoST-MH compared to eUC. We did not find that patient factors (ie, sex, race, college plans) or baseline mental health scores were significantly associated with self-efficacy.

### Mental Health Symptom Severity

**Figure 4:**
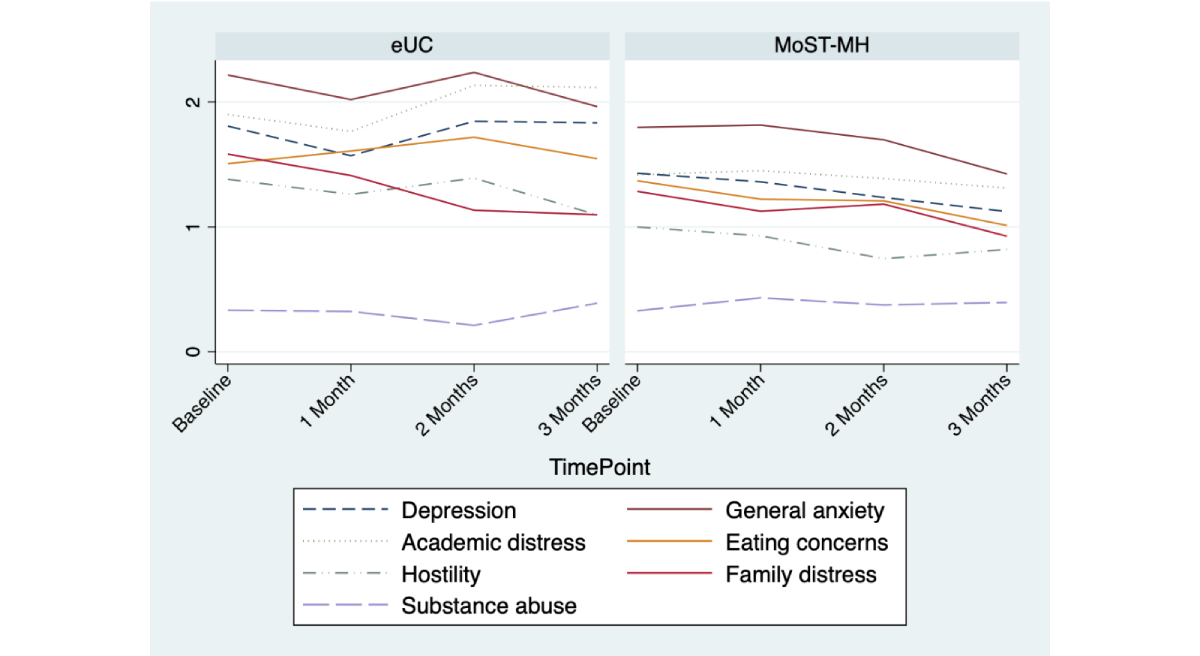
Change over time in College Counseling Center Assessment of Psychological Symptoms subscales. eUC: enhanced usual care; MoST-MH: Mobile Support Tool for Mental Health.

Using the CCAPS, in the MoST-MH arm, mental health symptom severity was reduced from baseline to 3 months in all subscales save substance abuse. In eUCs, reduced symptoms over 3 months occurred for general anxiety, family distress, and hostility only. Table 3 and Figure 4 show the mean scores on CCAPS subscales across treatment and time. In GEE analysis, there was a significant time effect such that at 1 month, depression scores were lower than baseline (beta=–.28, 95% CI –0.53 to –0.04) and time × treatment effect such that MoST-MH had lower depression scores relative to eUC by 3 months (beta=–0.34, 95% CI –0.67 to –0.03). In sensitivity analyses with imputed CCAPS outcome data, no significant effects of treatment were seen.

**Table 3:** Mental health symptoms over time by treatment.

CCAPS^a^ subscale		BL^b^ (eUC^c^ n=18, MoST-MH^d^ n=34), mean (SD)	1 m (eUC n=17, MoST-MH n=32), mean (SD)	2 m (eUC n=15, MoST-MH n=32), mean (SD)	3 m (eUC n=12, MoST-MH n=27), mean (SD)
**Depression**					
	eUC	1.81 (0.99)	1.57 (1.00)	1.85 (1.02)	1.83 (1.11)
	MoST-MH	1.43 (0.95)	1.36 (0.96)	1.24 (0.94)	1.12 (0.94)
**General anxiety**					
	eUC	2.22 (0.95)	2.02 (1.07)	2.24 (0.99)	1.96 (0.93)
	MoST-MH	1.82 (1.05)	1.82 (1.05)	1.70 (1.15)	1.42 (0.99)
**Social anxiety**					
	eUC	2.37 (0.93)	2.15 (0.95)	2.39 (1.02)	2.39 (0.90)
	MoST-MH	2.11 (0.93)	2.06 (1.07)	1.92 (1.07)	1.81 (1.03)
**Academic distress**					
	eUC	1.90 (0.88)	1.76 (0.96)	2.13 (0.90)	2.12 (1.11)
	MoST-MH	1.42 (1.02)	1.45 (1.06)	1.39 (1.06)	1.31 (0.98)
**Eating concerns**					
	eUC	1.51 (1.06)	1.61 (1.11)	1.72 (1.13)	1.55 (0.97)
	MoST-MH	1.37 (0.89)	1.22 (1.03)	1.21 (0.97)	1.01 (0.86)
**Family distress**					
	eUC	1.58 (0.81)	1.41 (1.00)	1.13 (0.83)	1.10 (0.71)
	MoST-MH	1.28 (0.85)	1.13 (0.96)	1.18 (0.82)	0.93 (0.63)
**Hostility**					
	eUC	1.38 (0.61)	1.26 (0.81)	1.39 (0.86)	1.1 (0.73)
	MoST-MH	1.00 (0.83)	0.93 (0.80)	0.75 (0.82)	0.82 (0.89)
**Substance abuse**					
	eUC	0.33 (0.72)	0.32 (0.54)	0.21 (0.38)	0.39 (0.76)
	MoST-MH	0.33 (0.60)	0.43 (0.72)	0.38 (0.60)	0.40 (0.58)

^a^CCAPS: College Counseling Center Assessment of Psychological Symptoms.

^b^BL: baseline.

^c^eUC: enhanced usual care.

^d^MoST-MH: Mobile Support Tool for Mental Health.

### Mental Health Care Services

In the month prior to enrollment, 56% of MoST-MH and 67% of eUC participants had received any mental health care. Over the first 3 months of enrollment, 52% of MoST-MH and 65% of eUC participants received any mental health care. Table 4 shows the percentage of participants who received inpatient, outpatient, and primary care for mental health in the prior month at each assessment point. In GEE analysis, there were no significant time, treatment, or time by treatment effect of MoST-MH compared to eUC.

**Table 4:** Mental health care over time by treatment.

Type of care		BL^a^ (eUC^b^ n=18, MoST-MH^c^ n=34), mean (SD)	M1 (eUC n=17, MoST-MH n=32), mean (SD)	M2 (eUC n=15, MoST-MH n=32), mean (SD)	M3 (eUC n=13, MoST-MH n=31), mean (SD)
**Any mental health care**					
	eUC	12 (67)	11 (65)	11 (73)	7 (54)
	MoST-MH	19 (56)	17 (53)	16 (50)	15 (48)
**Inpatient**					
	eUC	2 (11)	0	1 (7)	0
	MoST-MH	2 (6)	0	0	0
**Outpatient**					
	eUC	9 (50)	11 (65)	10 (67)	7 (54)
	MoST-MH	13 (38)	15 (47)	12 (38)	12 (39)
**Primary care**					
	eUC	4 (22)	1 (6)	3 (20)	1 (8)
	MoST-MH	9 (26)	4 (13)	6 (19)	5 (16)

^a^BL: baseline.

^b^eUC: enhanced usual care.

^c^MoST-MH: Mobile Support Tool for Mental Health.

## Discussion

### Principal Findings

In this pilot trial, we found evidence that, for young adults with mental health diagnoses transitioning to college, the MoST-MH intervention was engaged with at high rates, had high usability ratings, and may have reduced depression symptoms over time relative to the eUC group. Together, these findings provide initial support for an automated digital intervention incorporating periodic text message mental health check-ins that trigger web-based check-ins to understand stressors, negative effects, and self-efficacy and then provide self-efficacy support strategies.

Compared to other mental health digital interventions, we found good engagement:100% of text message check-ins and 78% of web-based check-ins were completed over 3 months, and no participant dropped out (ie, texted Quit). In contrast, among 28 digital mental health interventions for college students included in a prior systematic review [13], the average engagement rate was 56%. Complementing our finding high MoST-MH engagement, we found high usability ratings, suggesting the ease of use of text messages and web-based interfaces as well as the brief clear nature of support messaging.

We speculate that the high engagement and usability findings for MoST-MH can be attributed to several key design features. First, the study was introduced by a care provider, increasing trust in the intervention. Second, we designed MoST-MH with key input from a college student ambassador. Third, using text messaging to conduct most communication limited the amount of extra steps traditionally involved with apps or web pages, thus increasing ease of interaction. Fourth, the MoST-MH intervention adapted over time so that if an individual was not in need of support, the check-ins were stepped down (ie, occurred monthly), reducing the burden on individuals who seemed to not need help at that time.

The finding of reduced mental health symptoms in MoST-MH compared with eUC suggests that the intervention may successfully support self-management of mental health symptoms and that mood regulation may be a key mechanism. This is encouraging given the simplicity of the intervention and lack of any intensive cognitive behavioral treatment components. However, despite an intended aim of MoST-MH to enhance an individual’s self-efficacy, we did not find evidence to support this as a mechanism of action. Future studies of MoST-MH will be necessary to identify mechanisms of effects.

The use of mental health care decreased slightly in both treatment arms over time, yet it was still occurring in approximately half of all participants any given month. Also, there were no apparent signals of either increased or decreased mental health care use in the MoST-MH arm compared to eUC. On one hand, this is discouraging given that the MoST-MH intervention prompted many participants to reach out for mental health care when they reported low self-efficacy 2 weeks in a row. On the other hand, it may be that the MoST-MH intervention promoted confidence in self-management not reflected in the self-efficacy scales. Future studies are needed to identify how MoST-MH may modify cognitions around mental health care seeking.

### Limitations and Strengths

Several limitations should be discussed. First, we recruited mostly female White youths, therefore findings may not be valid in men or racial or ethnic minorities. Second, we did not follow participants for more than 3 months, limiting understanding of durability and prolonged engagement. Third, our cohort was recruited entirely during the COVID-19 pandemic. As such, the experience and stresses related to the transition to college and the college experience were atypical. Fourth, our sample is transdiagnostic, and we did not assess for specific mental health diagnoses or collect measures on type and other current treatments being received for mental health diagnoses, which may have been key moderators of effectiveness.

We note several strengths of our study and intervention design. First, we recruited individuals with any mental health diagnosis, thus rendering our intervention and findings broadly relevant to young adults transitioning to college (transdiagnostic). Second, we compared the MoST-MH intervention to eUC instead of a waitlist control, isolating effects of the digital interactions from those of attention and the psychoeducational videos. Third, we achieved high follow-up rates (86% at 3 months), reducing the likelihood of biased outcome analyses. Fourth, we measured and reported engagement through detailed analysis of reports through both text messages and web check-ins, notably absent in much prior digital MH intervention research [13]. Fifth, MoST-MH was completely automated, allowing low-cost scalability. Although human interaction has been identified as an important component of digital mental health interventions [24,25] and the majority of text message interventions for adolescent mental health and substance abuse involve some human communication [26], it is not feasible in the current reimbursement landscape to expect mental health care to fund these personnel. Digital intervention science should focus on identifying features to optimize human-computer interaction.

### Conclusions

We found preliminary evidence in support of an automated digital mental health intervention using periodic check-ins to tailor self-management support for youth with mental health disorders transitioning to college. This study is timely, as there is an urgent need for evidence-based mental health support programs for youth in transition to college that are used longitudinally and can be scaled easily. A program like MOST-MH, if found to be effective at reducing mental health symptoms and improving psychological functioning in a larger trial, could fill a needed gap in supporting youth in their mental health.

## Appendix

Multimedia Appendix 1 CONSORT-eHEALTH checklist (V 1.6.2).

## Abbreviations

CCAPS: College Counseling Center Assessment of Psychological Symptoms
C-SRI: Client Service Receipt Inventory for Mental Health
eUC: enhanced usual care
GEE: general estimating equation
MHSES: Mental Health Self-Efficacy Scale
MoST-MH: Mobile Support Tool for Mental Health
PSSUQ: Post-Study System Usability Scale
RCT: randomized controlled trial
