# Supplementary material for: Acceptability, Engagement, and Effects of a Mobile Digital Intervention to Support Mental Health for Young Adults Transitioning to College: Pilot Randomized Controlled Trial
Source: JMIR Form Res. 2021 Oct 14;5(10):e32271. doi: 10.2196/32271 (PMC8554670; doi:10.2196/32271)
Supplement: Multimedia Appendix 1 [file formative_v5i10e32271_app1.pdf]

CONSORT-EHEALTH Checklist V1.6.2 Report	Manuscript Number	32271

(based on CONSORT-EHEALTH V1.6), available at [http://tinyurl.com/consort-ehealth-v1-6].

Date completed
10/1/2021 14:17:57
by
Brian Suffoletto

TITLE

1a-i) Identify the mode of delivery in the title

1a-ii) Non-web-based components or important co-interventions in title
"Mobile Digital Intervention"
1a-iii) Primary condition or target group in the title
Does not pertain to this study

ABSTRACT

1b-i) Key features/functionalities/components of the intervention and comparator in the METHODS section of the ABSTRACT

1b-ii) Level of human involvement in the METHODS section of the ABSTRACT
"MoST-MH included periodic text-message and web-based check-ins of emotional health, stressors, negative impacts, and self-efficacy, which informed tailored self-care support messages. Both eUC and MoST-MH participants received links to a library of psycho-educational videos"
1b-iii) Open vs. closed, web-based (self-assessment) vs. face-to-face assessments in the METHODS section of the ABSTRACT
Does not pertain to this study
1b-iv) RESULTS section in abstract must contain use data
"were recruited from one primary care (n=31) and one mental health clinic (n=21)."
1b-v) CONCLUSIONS/DISCUSSION in abstract for negative trials
"Participants were randomized 2:1 to either receive MoST-MH (n=34) or enhanced Usual Care (eUC; n=18)."

INTRODUCTION

2a-i) Problem and the type of system/solution

2a-ii) Scientific background, rationale: What is known about the (type of) system
"MoST-MH was intended to provide support independent of mental health diagnosis type (i.e. trans-diagnostic) and designed to minimize burden of intensive digital interactions using a stepwise algorithm which adapts frequency of interaction to the needs of the youth."

METHODS

3a) CONSORT: Description of trial design (such as parallel, factorial) including allocation ratio
There has to be a better and more efficient way. This is way too burdensome.
3b) CONSORT: Important changes to methods after trial commencement (such as eligibility criteria), with reasons
"We hypothesized that youth would engage with MoST-MH at high rates over the first 3-months of college and that they would report high levels of usability. We also hypothesized that youth who received MoST-MH, as compared with youth who receive eUC, would report greater mental health self-efficacy, lower symptom severity, and higher rates of follow-through with mental health care at 3-months."
3b-i) Bug fixes, Downtimes, Content Changes

4a) CONSORT: Eligibility criteria for participants
"We conducted a pilot randomized trial among youth with a current mental health disorder and/or recent mental health care preparing to transition to college. Design and a priori hypotheses were registered (clinicaltrials.gov NCT04560075). As this was a pilot study, we were not powered to detect significant differences in mental health outcomes between groups. All participants completed written informed consent. Study investigators and outcome assessors were blinded to allocation to treatment arms."
4a-i) Computer / Internet literacy

4a-ii) Open vs. closed, web-based vs. face-to-face assessments:
Not applicable to this study
4a-iii) Information giving during recruitment
"Participants were recruited from one primary care (n=31) and one mental health clinic (n=21) in Pittsburgh, PA from August to October, 2020"
4b) CONSORT: Settings and locations where the data were collected
Not applicable to this study
4b-i) Report if outcomes were (self-)assessed through online questionnaires

4b-ii) Report how institutional affiliations are displayed
"Each monthly assessment battery was estimated to take 15 minutes to complete and were completed on a smartphone, laptop, tablet or desktop. Participants in both groups were sent text-message reminders every 3 days up to 3 times prompting them to complete their web-based follow-up assessment batteries."
5) CONSORT: Describe the interventions for each group with sufficient details to allow replication, including how and when they were actually administered
5-i) Mention names, credential, affiliations of the developers, sponsors, and owners

5-ii) Describe the history/development process
"we developed an automated Mobile Support Tool for Mental Health (MoST-MH). MoST-MH was iteratively designed and refined by a multi-disciplinary team with expertise in psychology, psychiatry, primary care, and digital interventions with integral feedback from a college student ambassador."
5-iii) Revisions and updating
We had not done any prior usability testing
5-iv) Quality assurance methods
Not applicable to this study
5-v) Ensure replicability by publishing the source code, and/or providing screenshots/screen-capture video, and/or providing flowcharts of the algorithms used
There was no specific quality assurance set up
5-vi) Digital preservation

"Upon allocation, MoST-MH participants were prompted to text a unique keyword to our study phone number to initiate the program. Once initiated, participants received a series of welcome messages describing what to expect over the intervention period and ways to reduce breach of privacy. For example: "Welcome to MoST-MH. Over the next 3 months we'll be checking in by text message. Set up a password on your phone and erase messages you do not want anyone to see after reading them." Participants were instructed that they can drop out of the MoST-MH program at any time by texting "Quit."<br>Starting on the day of enrollment, MoST-MH participants received mental health check-in via text message: "How would you rate your emotional health this past week?". If they replied "excellent, very good or good", they received a positive feedback text message and link to video library. The brief 2-minute videos were created by the study team and included psycho-education about mental health self-care during college. If they replied "fair" or "poor", they were sent a link to complete a web-based check-in. Upon opening the web-link, a page displayed a checklist of common stressors7 and negative effects (shown in Table 2). Then they were asked a self-efficacy question: "To what extent do you feel you can manage your stressors and negative effects with supports and skills you have?" If they reported high self-efficacy ("completely"), they received positive feedback, a web-link to a library of mental health videos, and the program was timed to check in with them in a month.<br>If they reported low self-efficacy ("somewhat"; "a little"; or "not at all"), they received a text message from a skills library, the link to the videos, and were asked if it was ok to check in next week. On subsequent MoST-MH check-ins, their reports of stressors and negative effects were compared to the prior assessment, and feedback incorporated relative improvement or unresolved stressors/effects. If the ability to self-manage stressors or negative effects was still reported as sub-optimal, the individual was prompted to consider making an appointment for seeking mental health care: "Your doctor or another health professional can help. Would you be willing to reach out to them to set up an appointment?" If they were willing, they were provided with a link to resources to assist. Throughout all program queries, missing responses were re-prompted once only. To ensure safety, if an individual reported poor mental health and low self-efficacy 2 weeks in a row, they were prompted to seek formal MH care. Figure 2 demonstrates an example communication exchange. "		
**5-vii) Access**		
See prior response. All content represented in figures and Methods.		
**5-viii) Mode of delivery, features/functionalities/components of the intervention and comparator, and the theoretical framework**		
See Section V above for further description		
**5-ix) Describe use parameters**		
"MoST-MH was intended to provide support independent of mental health diagnosis type (i.e. trans-diagnostic) and designed to minimize burden of intensive digital interactions using a stepwise algorithm which adapts frequency of interaction to the needs of the youth. MoST-MH is an ecological momentary intervention (EMI)16 in that it provides support in the context of a young adult's current state and needs. Specifically, MoST-MH incorporated periodic text-message mental health check-ins, which triggered web-based check-ins (when mental health is rated low) to understand stressors, negative effects, and self-efficacy, which informed self-efficacy support strategies and prompted links to psychoeducational videos focused on college and mental health. Figure 1 outlines the design of MoST-MH. "		
**5-x) Clarify the level of human involvement**		
See above Section V for complete description		
**5-xi) Report any prompts/reminders used**		
Not applicable to our study		
**5-xii) Describe any co-interventions (incl. training/support)**		
See above Section V for complete description		
**6a) CONSORT: Completely defined pre-specified primary and secondary outcome measures, including how and when they were assessed**		
"Participants were recruited from one primary care (n=31) and one mental health clinic (n=21) in Pittsburgh, PA from August to October, 2020. We chose to recruit from healthcare sites because we view the ultimate implementation to be initiated by care providers who are able to identify individuals with mental health needs prior to leaving for college. The youth's care provider(s) identified potentially eligible youth and asked the youth about interest in participating in the study; interested youth were texted or emailed a web link that provided information about the study. If they were interested, they contacted the research team via telephone, where enrollment criteria were confirmed. Inclusion criteria included: 18 years of age or older, current mental health diagnosis documented in their electronic medical record and/or in receipt of mental health services within 3 months per self-/ parent- or clinician- report, graduated high school, plan to attend college or higher education within 6 weeks, and own a personal mobile phone with text messaging. We excluded non-English-speaking individuals given that intervention materials were in English only. "		
**6a-i) Online questionnaires: describe if they were validated for online use and apply CHERRIES items to describe how the questionnaires were designed/deployed**		
**6a-ii) Describe whether and how "use" (including intensity of use/dosage) was defined/measured/monitored**		
"MoST-MH usability was measured via the Post-Study System Usability Scale (PSSUQ)19 at 3-months only. The PSSUQ includes 19 items, each rated on a 7-point Likert-type scale ranging from 1 (strongly agree) to 7 (strongly disagree). The psychometric factors of the PSSUQ are (1) overall usability, (2) system usefulness, (3) information quality, and (4) interface quality. The lower the score (to a limit of one), the higher the perceived usability.<br>Mental Health Self-Efficacy was measured using the 6-item self-report Mental Health Self-Efficacy Scale (MHSES)20, which asks participants to rate each statement on a 10-point Likert scale ranging from 1 ("Not at all confident") to 10 ("Totally confident") whereby higher scores indicate higher self-efficacy: "On an average day in the next month, how confident are you that… (1) You can keep your stress, anxiety or depression from interfering with the things that you want to do?; (2) You can do the different tasks and activities needed to manage your stress, anxiety or depression so as to reduce your need to see a doctor?; (3) You can do things other than just taking medicine to reduce how much your stress, anxiety or depression affects your everyday life?; (4) You can make your days at least moderately enjoyable?; (5) You will have moderate amounts of time where you do not experience stress, anxiety or depression?; and (6) You will be able to effectively manage any stress, anxiety or depression that you do experience?"<br>Symptom Severity was measured using the College Counseling Center Assessment of Psychological Symptoms (CCAPS)21 which has 62 items with eight distinct subscales of psychological symptoms for college students: (a) Depression (13 items), (b) Generalized Anxiety (9 items), (c) Social Anxiety (7 items), (d) Academic Distress (5 items), (e) Eating Concerns (9 items), (f) Family Distress (6 items), (g) Hostility (7 items), and (h) Substance Use (6 items). Items are scored on a 5-point Likert scale from 0 ("Not at all like me") to 4 ("Extremely like me"), whereby higher scores indicate higher symptom severity.<br>Mental Health Treatment Utilization was measured using the brief self-report Client Service Receipt Inventory for Mental Health (C-SRI)22 including outpatient, inpatient, and medication management services."		
**6a-iii) Describe whether, how, and when qualitative feedback from participants was obtained**		
"We tested the hypothesis that youth would engage with MoST-MH at high rates (>80% response rate) by calculating text message and web check-in completions within and between individuals."		
**6b) CONSORT: Any changes to trial outcomes after the trial commenced, with reasons**		
"Each monthly assessment battery was estimated to take 15 minutes to complete and were completed on a smartphone, laptop, tablet or desktop. Participants in both groups were sent text-message reminders every 3 days up to 3 times prompting them to complete their web-based follow-up assessment batteries."		
**7a) CONSORT: How sample size was determined**		
**7a-i) Describe whether and how expected attrition was taken into account when calculating the sample size**		
**7b) CONSORT: When applicable, explanation of any interim analyses and stopping guidelines**		
"We tested the hypothesis that youth would engage with MoST-MH at high rates (>80% response rate) by calculating text message and web check-in completions within and between individuals. We tested the hypothesis that youth would report high levels of usability with MoST-MH (mean PSSUQ rating <=2) by computing PSSUQ ratings at 3-month follow-up. We explored the effect of MoST-MH, as compared with eUC, on mental health self-efficacy (MHSES), symptom severity (CCAPS), and mental healthcare services utilization (C-SRI) using mixed-effect (general estimating equation: GEE) models. Mixed-effects models using GEE are recommended for analysis of repeated-measures data and can properly account for missing data.23 To understand for whom the intervention may works better/worse for, we explored associations between patient factors (sex, race, planned college attendance, baseline CCAPS scores) and engagement, usability, and mental health outcomes using univariate GEE models. Primary analyses were conducted using listwise deletion."		
**8a) CONSORT: Method used to generate the random allocation sequence**		
Not applicable to this study		
**8b) CONSORT: Type of randomisation; details of any restriction (such as blocking and block size)**		
We did not stop trial or conduct interim analyses		
**9) CONSORT: Mechanism used to implement the random allocation sequence (such as sequentially numbered containers), describing any steps taken to conceal the sequence until interventions were assigned**		

"We used block randomization whereby 2/3 of participants were randomly assigned to receive MoST-MH, and 1/3 to receive eUC. Blocks balanced the groups based on recruitment site. Random assignment allocation occurred following completion of baseline assessments."		
**10) CONSORT: Who generated the random allocation sequence, who enrolled participants, and who assigned participants to interventions**		
"We used block randomization whereby 2/3 of participants were randomly assigned to receive MoST-MH, and 1/3 to receive eUC. Blocks balanced the groups based on recruitment site. Random assignment allocation occurred following completion of baseline assessments."		
**11a) CONSORT: Blinding - If done, who was blinded after assignment to interventions (for example, participants, care providers, those assessing outcomes) and how**		
**11a-i) Specify who was blinded, and who wasn't**		
**11a-ii) Discuss e.g., whether participants knew which intervention was the "intervention of interest" and which one was the "comparator"**		
It was not possible to blind participants in this trial. Investigators were blinded to treatment assignment.		
**11b) CONSORT: If relevant, description of the similarity of interventions**		
Randomization sequence was blinded to investigators		
**12a) CONSORT: Statistical methods used to compare groups for primary and secondary outcomes**		
"Trial registration: clinicaltrials.gov NCT04560075" Otherwise full protocol resides with NIH.		
**12a-i) Imputation techniques to deal with attrition / missing values**		
**12b) CONSORT: Methods for additional analyses, such as subgroup analyses and adjusted analyses**		
" The eUC participants received a web link to a library of the same psycho-educational videos provided to the MoRE-MH group. eUC received no text message or web-based MH check-ins."		
RESULTS		
**13a) CONSORT: For each group, the numbers of participants who were randomly assigned, received intended treatment, and were analysed for the primary outcome**		
Not applicable to this study		
**13b) CONSORT: For each group, losses and exclusions after randomisation, together with reasons**		
See above. No further sub-group analyses conducted		
**13b-i) Attrition diagram**		
**14a) CONSORT: Dates defining the periods of recruitment and follow-up**		
"Figure 3 shows the participant flow throughout the study. A total of 98 youth were referred to the study, 73 were reached for screening, and 52 youth completed informed consent. The resultant sample was randomized via computer algorithm to receive either instructions to initiate MoST-MH (n=34) or eUC (n=18) after completion of online questionnaires at baseline. 93% of participants completed 1-month follow-up assessment batteries, 88% completed 2-month follow-up assessment batteries and 74% completed 3-month follow-up assessment batteries"		
**14a-i) Indicate if critical "secular events" fell into the study period**		
**14b) CONSORT: Why the trial ended or was stopped (early)**		
"Participants were sent a median of 5 (range 3-10) text-message check-ins over the study period (depending on their risk level) and 100% of the text-message queries were completed. The 21 participants who reported poor MH via text message at least once over the study period received a median of 2 (range 1 to 8) web-based check-ins. Of the 55 times when a web check-in was prompted, 43 (78%) were completed. We did not find that sex, race, or college living plans were significantly associated with web check-ins. No MoST-MH participant dropped-out (i.e. texted "quit"). "		
**15) CONSORT: A table showing baseline demographic and clinical characteristics for each group**		
"Participants were recruited from one primary care (n=31) and one mental health clinic (n=21) in Pittsburgh, PA from August to October, 2020."		
**15-i) Report demographics associated with digital divide issues**		
**16a) CONSORT: For each group, number of participants (denominator) included in each analysis and whether the analysis was by original assigned groups**		
**16-i) Report multiple "denominators" and provide definitions**		
**16-ii) Primary analysis should be intent-to-treat**		
"Participants were sent a median of 5 (range 3-10) text-message check-ins over the study period (depending on their risk level) and 100% of the text-message queries were completed. The 21 participants who reported poor MH via text message at least once over the study period received a median of 2 (range 1 to 8) web-based check-ins. Of the 55 times when a web check-in was prompted, 43 (78%) were completed. We did not find that sex, race, or college living plans were significantly associated with web check-ins. No MoST-MH participant dropped-out (i.e. texted "quit").<br>The median number of stressors reported per check-in was 2 (range 0 to 5); the median number of negative effects reported per check-in was 3 (range 0 to 8). There were higher mean stressors reported in female compared to male participants (beta=0.49; 95% CI 0.21, 0.76). The most common stressors were related to school and finances; the most common negative effects were feeling worn out and low motivation. Table 2 shows the percentage of check-ins with a given stressor and negative effects reported. Self-management self-efficacy was rated as high 9.3% of the time and low 90.7% of the time. We did not find that patient factors (i.e. sex, race, college plans) were significantly associated with self-efficacy. Higher baseline anxiety scores were associated with lower self-efficacy (beta=-0.32; 95% CI -0.55, -0.08)."		
**17a) CONSORT: For each primary and secondary outcome, results for each group, and the estimated effect size and its precision (such as 95% confidence interval)**		
The trial ended when we met recruitment goals for the pilot		
**17a-i) Presentation of process outcomes such as metrics of use and intensity of use**		
**17b) CONSORT: For binary outcomes, presentation of both absolute and relative effect sizes is recommended**		
See Table 1 for further description		
**18) CONSORT: Results of any other analyses performed, including subgroup analyses and adjusted analyses, distinguishing pre-specified from exploratory**		
"Using the CCAPS, in the MoST-MH arm, mental health symptom severity was reduced from baseline to 3-months in all sub-scales save substance abuse. In eUCs, reduced symptoms over 3-months occurred for general anxiety, family distress, and hostility only. Table 4 and Figure 3 shows the mean scores on CCAPS sub-scales across treatment and time. In GEE analysis, there was a significant time effect such that at 1-month, depression scores were lower than baseline (beta=-.28; 95% CI -0.53 to -0.04) and time x treatment effect such that MoST-MH had lower depression scores relative to eUC by 3-months (beta=-0.34; 95% CI -0.67 to -0.03). In sensitivity analyses with imputed CCAPS outcome data, no significant effects of treatment were seen"		
**18-i) Subgroup analysis of comparing only users**		
**19) CONSORT: All important harms or unintended effects in each group**		
See above for further description		
**19-i) Include privacy breaches, technical problems**		
**19-ii) Include qualitative feedback from participants or observations from staff/researchers**		
No privacy breaches occured.		
DISCUSSION		
**20) CONSORT: Trial limitations, addressing sources of potential bias, imprecision, multiplicity of analyses**		
**20-i) Typical limitations in ehealth trials**		
**21) CONSORT: Generalisability (external validity, applicability) of the trial findings**		
**21-i) Generalizability to other populations**		
**21-ii) Discuss if there were elements in the RCT that would be different in a routine application setting**		
See limitations for further description		

**22) CONSORT: Interpretation consistent with results, balancing benefits and harms, and considering other relevant evidence**		
**22-i) Restate study questions and summarize the answers suggested by the data, starting with primary outcomes and process outcomes (use)**		
**22-ii) Highlight unanswered new questions, suggest future research**		
"In this pilot trial, we found evidence that, for young adults with mental health diagnoses transitioning to college, an automated mobile support tool for mental health (MoST-MH) is engaged with at high rates, has high usability ratings, and reduces depression symptoms over time relative to an enhanced care eUC group. Together, these findings provide initial support for an automated digital intervention incorporating periodic text-message mental health check-ins, which triggers web-based check-ins to understand stressors, negative effects, and self-efficacy, which then informs self-efficacy support strategies."		
Other information		
**23) CONSORT: Registration number and name of trial registry**		
Not applicable to this study		
**24) CONSORT: Where the full trial protocol can be accessed, if available**		
"In the month prior to enrollment, 56% of MoST-MH and 67% of eUC participants had received any mental healthcare. Over the first 3 months of enrollment, 52% of MoST-MH and 65% of eUC participants received any mental healthcare. Table 5 shows the percentage of participants who received inpatient, outpatient, and primary care for mental health in the prior month at each assessment point. In GEE analysis, there were no significant time, treatment, or time by treatment effect of MoT-MH compared to eUC."		
**25) CONSORT: Sources of funding and other support (such as supply of drugs), role of funders**		
"Trial registration: clinicaltrials.gov NCT04560075"		
**X26-i) Comment on ethics committee approval**		
**x26-ii) Outline informed consent procedures**		
"All procedures were approved by the Institutional Review Board at the University of Pittsburgh."		
**X26-iii) Safety and security procedures**		
"All participants completed written informed consent."		
**X27-i) State the relation of the study team towards the system being evaluated**		
